# Trends in nationwide incidence of pediatric type 1 diabetes in Montenegro during the last 30 years

**DOI:** 10.3389/fendo.2022.991533

**Published:** 2022-09-06

**Authors:** Maja Raicevic, Mira Samardzic, Ivan Soldatovic, Natasa Curovic Popovic, Rade Vukovic

**Affiliations:** ^1^ Department of Endocrinology, Institute for Children’s Diseases, Clinical Centre of Montenegro, Podgorica, Montenegro; ^2^ Faculty of Medicine, University of Montenegro, Podgorica, Montenegro; ^3^ Institute of Medical Statistics and Informatics, Faculty of Medicine, University of Belgrade, Belgrade, Serbia; ^4^ Department of Endocrinology, Mother and Child Health Care Institute of Serbia “Dr Vukan Cupic”, Belgrade, Serbia; ^5^ School of Medicine, University of Belgrade, Belgrade, Serbia

**Keywords:** diabetes mellitus, T1D, incidence, COVID19, Montenegro (Crna Gora)

## Abstract

Significant and unexplained variations in type 1 diabetes (T1D) incidence through the years were observed all around the world. The update on this disorder’s incidence is crucial for adequate healthcare resource planning and monitoring of the disease. The aim of this study was to give an update on the current incidence of pediatric T1D in Montenegro and to analyze incidence changes over time and how the exposure to different factors might have affected it. This retrospective cohort study included a total of 582 patients younger than 15 years who were newly diagnosed with T1D during the past 30 years. The average age at diagnosis was 8.4 ± 3.91 years. The mean annual incidence of T1D in the Montenegro population during the whole study period of 30 years was 15.2/100,000 person-years. Slightly higher incidence rates were observed in male compared to female individuals, and the incidence increased with age, with the highest incidence in the 10–14 age group. If the model is observed as one without jointpoints, the annual percentage change (APC) for the total population is 3.1 (1.8–4.4); for male individuals, 3.8 (2.1–5.5); and for female individuals, 2.1 (0.6–3.5). In 2020, the first year of the coronavirus disease of 2019 (COVID-19) pandemic, in comparison to 2019, the incidence rate increased from 19.7/100,000 to 21.5/100,000, with the highest increase in the age group of 5–9 years. This is the first nationwide report on a 30-year period of T1D incidence trend in Montenegro. It suggests that T1D incidence among Montenegrin children is rising again and that there is a short-term influence of COVID-19 on new-onset T1D.

## Introduction

Significant and unexplained variations in type 1 diabetes (T1D) incidence through the years were observed all around the world. The update on T1D incidence is crucial for unexceptional healthcare resource planning and monitoring of this disease, which is still unpreventable and concerning. Moreover, the incidence of T1D shows an increasing trend in Europe, and it sharply increased since 2019 (1).

North European countries (Finland, Sweden, Norway, the United Kingdom, and Ireland) are at the top of the list among countries with the highest incidence of T1D (27.5–52.2/100,000), but the incidence is also very high in the Italian region of Sardinia (45.0/100,000), which is located in the south-western part of Europe (1, 2).

According to the last census (2011), Montenegro is a south-eastern European country, with an area of 13,812 km^2^ and a population of 620,145, including 118,751 (19%) children younger than 15. Results of prior studies classified Montenegro as a country with a high incidence rate of T1D (18.5/100,000 for the period 2009–2013) (3, 4). However, the last report regarding the incidence of T1D was based on data from almost a decade ago, and analysis of more recent data is needed to gain insight into the current epidemiological situation regarding T1D in Montenegro.

The aim of this study was to give an update on the current incidence of pediatric T1D in Montenegro and to analyze incidence changes over time and how the exposure to different factors might have affected it, which should be useful for further projection of T1D prevalence.

## Methods

The study included children younger than 15 years who were newly diagnosed with T1D during the period 1991–2020. The data source was the Diabetes Registry of Montenegro (established according to the EURODIAB Collaborative group propositions) and patients’ medical records at the Institute for Children’s Diseases, Clinical Center of Montenegro. The Institute for Children’s Diseases is the referent diabetes center for Montenegro, the only center where the diagnosis of T1D could be made and where insulin therapy is initially started and prescribed. Study protocol was formally approved by the Ethics Committee of Clinical Center of Montenegro (no. 03/01-24708). Second data source were records from the Institute of Public Health of Montenegro, which provided capture–recapture methodology (5). Children with other types of diabetes, such as type 2 diabetes or maturity-onset diabetes of the youth (MODY) were excluded from the study. The population denominator data were obtained from the 1991, 2003, and 2011 national census data of the Central Bureau of Statistics (Monstat). Due to long intervals between national censuses, the estimated numbers of habitants were used in order to obtain the number of habitants for each year in the period between 2000 and 2020. In the period from 1991 to 1999, no estimates of population were found, and due to the fact that during these years, there was a conflict in the region and subsequently high population migration, modeling of data was used to obtain estimates of the population in this period. For that purpose, the polynomial regression analysis was used to fit the data and impute the missing values.

Collected variables included gender, date of birth, and date of the onset of T1D. Three types of incidence rates, expressed as new cases per 100,000 persons, were calculated: age specific, age standardized, and crude. Age-specific rates were adjusted to three age groups (0–4, 5–9, and 10–14). Age-adjusted incidence rates were calculated using Segi’s World population (6).

The annual percentage change (APC), a number assumed as a constant percentage change of the previous year rate, was determined using jointpoint regression analysis. The joinpoint analysis was performed in Joinpoint Regression Program, v4.9.0.0, March 2021, Statistical Research and Applications Branch, National Cancer Institute.

## Results

During the study period (1991–2020), there were a total of 582 children with newly diagnosed T1D, in which 317 were boys and 265 were girls. The average age at diagnosis was 8.4 ± 3.91 years. The mean annual incidence of T1D in the Montenegro population during the whole study period of 30 years was 15.2/100,000 person-years. The age- and sex-category-specific rates for the whole study period are shown in **Table 1** and age-standardized incidence rate for 5-year period in **Table 2**.

**Table 1:** Number of cases, total person years, means annual incidence, age-specific incidence, and age-standardized incidence of T1D in Montenegro during the period 1991–2020.

	No. of cases	Total person years	Mean annual age spec. incidence (95% CI) per 100,000	Age stand. incidence per 100,000
**Total**				
** All (0–14)**	582	3,729,405	15.6 (1.44–4.69)	15.2
** 0–4**	133	1,201,286	11.1 (0.93–1.31)	
** 5–9**	214	1,223,417	17.5 (1.52–2.00)	
** 10–14**	235	1,304,702	18.0 (1.58–2.05)	
**Boys**				
** All (0–14)**	317	1,933,557	16.4 (1.46–1.83)	15.9
** 0–4**	70	625,164	11.2 (0.87–1.41)	
** 5–9**	113	633,132	17.9 (1.47–2.15)	
** 10–14**	134	675,261	19.8 (1.66–2.35)	
**Girls**				
** All (0–14)**	265	1,795,848	14.8 (1.30–1.66)	14.4
** 0–4**	63	576,122	10.9 (0.84–1.39)	
** 5–9**	101	590,285	17.1 (1.39–2.08)	
** 10–14**	101	629,441	16.1 (1.31–1.95)	

**Table 2:** Age-standardized incidence rates of T1D in children aged 0–14 in Montenegro—5-year periods compared.

Age std. inc. rate (per 100,000)	Total	Boys	Girls
**1991–1995**	7.6	7.1	8.12
**1996–2000**	12.1	12.1	12.1
**2001–2005**	14.8	11.4	18.5
**2006–2010**z	18.4	22.7	13.7
**2011–2015**	20.7	22.5	18.7
**2016–2020**	19.2	21.6	16.5
**Whole period**	15.2	15.9	14.4

**Figure 1:**
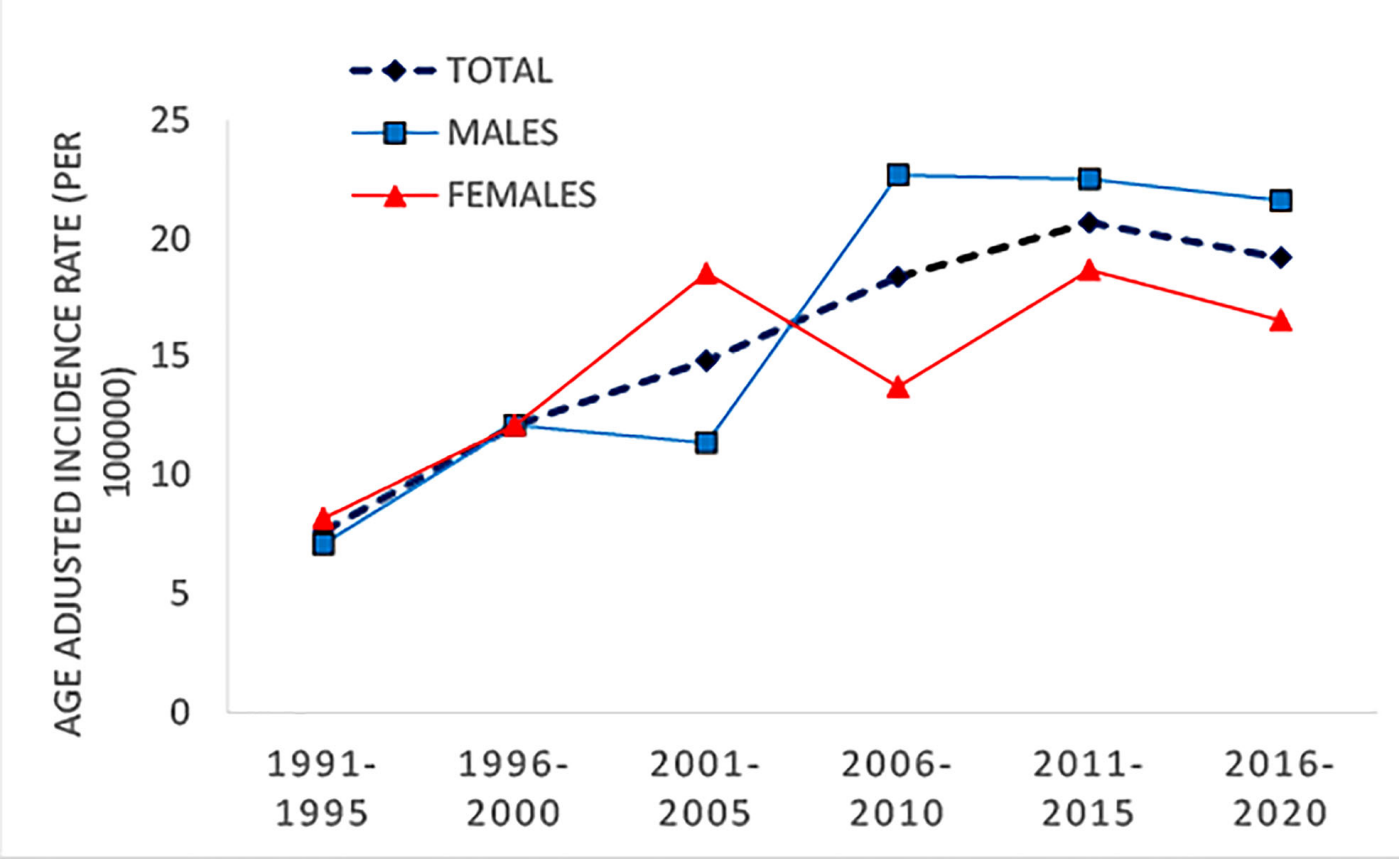
Trend of age-adjusted incidence rate (per 100,000), Montenegro, 1991–2020.

**Figure 2:**
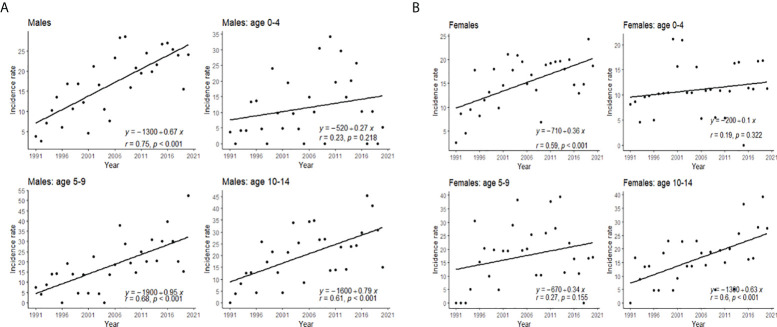
Trends of crude incidence rates of T1D in different age groups in **(A)** boys and **(B)** girls.

Slightly higher incidence rates were observed in male compared to female patients (**Figure 1**). The incidence increased with age, with the highest incidence in the 10–14 age group (**Figure 2**). The same trend is observed in the gender stratum (**Table 1**).

**Figure 3:**
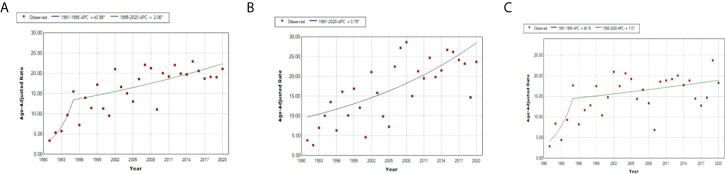
Jointpoint regression analysis: trend of standardized incidence rates of T1D in different gender groups, in **(A)** total, **(B)** boys, and **(C)** girls.

If the model is observed as one without jointpoints, the APC for the total population is 3.1 (1.8–4.4); for male individuals, 3.8 (2.1–5.5); and for female individuals, 2.1 (0.6–3.5). However, in both genders model, one significant joinpoint was obtained; for the period 1991–1995, APC is 40.96, while in the period 1995–2020, APC is 20.06. In female individuals, there is one significant joinpoint as well, and the APC for the period 1991–1995 is 36.76, while for the period 1995–2020, APC is 1.07. Every segment is shown separately in **Figure 3**.

In 2020, the first year of the COVID-19 pandemic, in comparison to 2019, the incidence rate increased from 19.7/100,000 to 21.5/100,000, with the highest increase in the age group of 5–9 years (**Table 3**), with similar variations among age and gender groups also reported at different time points during the past three decades.

**Table 3:** Crude incidence rate of T1D in Montenegro for the last 5 years, in different age groups.

Age groups (years)	0–4	5–9	10–14	Total
Crude incidence rate(per 100,000 persons/year)				
**2016**	10.9	28.6	23.1	21.0
**2017**	5.4	21.0	31.6	19.4
**2018**	13.4	10.6	34.9	19.6
**2019**	8.1	16.0	34.9	19.7
**2020**	8.2	35.4	21.1	21.5

## Discussion

The results of this nationwide epidemiological study in 0–14-year-old children showed that Montenegro is a country with a high incidence rate of T1D (in the range of 10–19.9/100,000) (7, 8).

The previous report on the incidence of T1D in Montenegro has been published by Patterson et al., in their multicentric study, and our results point out that the incidence is higher since then (3). If we observe only the last 15-year period, the incidence of T1D in Montenegro can be classified as very high in male individuals younger than 15 (21.6–22.7/100,000). Knowing the data on T1D incidence trend, in the whole 0–14-year-old population and also in different age subgroups, is very important for the monitoring of the disease and for adequate healthcare resource planning and distribution.

In comparison to neighboring countries, T1D incidence in Montenegro is similar to Serbia (14.3/100,000 for the period 2007–2017), Slovenia (16.3/100,000 for the period 2009–2013), and Croatia (17.2/100,000 for the period 2004–2012), but it is significantly higher than in a Former Yugoslavian Republic of Macedonia (7.7/100,000 for the period 2009–2013) and Bosnia and Herzegovina–Tuzla region (6.9/100,000 for the period 1990–1998), which have higher rates of incidence increase in the later period than in previous countries (possibly due to prior underreporting of T1D) (3, 9–11).

During the 30 years of follow-up, the incidence increased by approximately 3.1% annually, but in the last years, the increase appears to be slowing down. The suggested slowing in the period 2004–2008 and cyclical 5-year periodicity in incidence pattern were not observed in our country (3).

Similarly across Europe, T1D is predominantly diagnosed in children 10–14 years old, but the average age at the onset of the disease is a bit lower (3, 12, 13). It could be due to the high childhood obesity rate or lower immunization coverage rate, which will be discussed in the following text. Opposite to other studies, we did not observe the highest incidence rate increase in boys younger than 5, but it is less marked in girls aged 0–4 years than in other age/sex subgroups (3, 14). In 2020, the highest incidence rate increase was among 5–9 years old.

Significant variations in T1D incidence through the years are still unexplained. Although, genetics, lifestyle, and socioeconomic factors play important roles in the development of type 1 diabetes, a majority of studies suggest environmental factors as crucial (15–17). The emphasis is on the decrease in infectious diseases frequency, increase in vaccination coverage rate, and changes in the food and supplements intake.

According to the accelerator hypothesis, childhood obesity significantly impacts the development and incidence of all types of diabetes, including T1D (18). In both obesity and type 1 diabetes, leptin, resistin, and β-cell autoimmunity are elevated, but it is not clear yet if obesity accelerates or causes type 1 diabetes (19). It is known that, in 2013, every fourth Montenegrin school-aged child was overweight, and the number of overweight boys was twice higher in comparison to girls (20). Since then, many inhabitants from the rural parts migrated to the urban parts of the country; a lot of people started a sedentary lifestyle with increased intake of high-calorie foods, which is expected to have led to even higher obesity rate and sustained increase in T1D incidence.

Like the rest of the world, Montenegro has been affected by the ongoing worldwide pandemic (COVID-19 pandemic) caused by the severe acute respiratory syndrome coronavirus 2 (SARS-CoV-2) virus since its first case was confirmed on 17 March 2020. For more than 50 years, viruses are considered potential triggers for autoimmune diseases such as T1D (21, 22). Furthermore, some authors reported an increased incidence of new-onset T1D during the first year of the COVID-19 pandemic (23–25). SARS-CoV-2 tropism to pancreatic β cells is supposed to be due to their angiotensin-converting enzyme 2 (ACE2) receptors and neuropilin 1 (NRP1) receptors, and virus–receptor interactions lead to cell damage and impaired insulin secretion. Moreover, high blood glucose level “stimulates” replication of the virus and damage progress (26–28). Opposed to those findings and in line with our results, Mameli et al. have described the double wave occurrence, with the decrease in T1D incidence in the first wave of the COVID-19 pandemic, as also Kostopoulou reported (29, 30). It could be related to the fact that T1D is manifested a few months after a child’s contact with the trigger, in this case, the SARS-CoV-2 virus. On the other hand, if we observe a pandemic and strict lockdown in the first months as a stress, which could also be a trigger for T1D development, the incidence increase could be registered earlier (31). The highest increase in incidence rate during the first year of COVID-19 was observed in children older than 5 but younger than 10, which was also described as a finding in the Italian region of Calabria (13).

Hence, a similar peak in incidence rate to the one in the first year of the COVID pandemic was observed in 2016 (**Table 3**), which could be due to a significantly decreased rate of immunization with measles–mumps–rubella (MMR) vaccine among Montenegrin children that year (2015—whole country coverage of 93.5%, 98% in the capital city; 2016—whole country coverage of 86.4%, 73.0% in the capital city; 2017—whole country coverage of 92.2%, 97.7% in the capital city) (32–35). In the following years, the government introduced mandatory immunization certificates for children who want to stay in kindergarten, which improved the immunization coverage rate.

The strength of this study is its nationwide character and timeliness with a long observational period of 30 years supported by the completeness of the diabetes registry and high-quality data from the second source. The limitation of the present study is a lack of the exact number of inhabitants in the period 1991–1999, which we assumed and predicted using the mathematical mode (polynomial regression).

This is the first nationwide report on a 30-year period of T1D incidence trend in Montenegro. It suggests that T1D incidence among Montenegrin children is rising again, after a plateau, and that there is a short-term influence of COVID-19 on new-onset T1D. The highest increase in the first year of the COVID pandemic is registered in 5–9-year-olds.

## Data availability statement

The raw data supporting the conclusions of this article will be made available by the authors, without undue reservation.

## Author contributions

MR, MS, and RV designed the research study. MR and NP gathered the data. IS conducted statistical analyses. MR and IS wrote the first draft of the manuscript. All authors contributed to study design and revised and approved the final version of the manuscript.

## Conflict of interest

The authors declare that the research was conducted in the absence of any commercial or financial relationships that could be construed as a potential conflict of interest.

## Publisher’s note

All claims expressed in this article are solely those of the authors and do not necessarily represent those of their affiliated organizations, or those of the publisher, the editors and the reviewers. Any product that may be evaluated in this article, or claim that may be made by its manufacturer, is not guaranteed or endorsed by the publisher.

## Abbreviations

T1D, type 1 diabetes; EURODIAB, a collaboration of European Childhood Diabetes Registers; MODY, maturity onset diabetes of youth; APC, annual percentage change; COVID-19, coronavirus disease of 2019.
